# Studies on Pure Mlb^®^ (Multiple Left Border) Technology and Its Impact on Vector Backbone Integration in Transgenic Cassava

**DOI:** 10.3389/fpls.2022.816323

**Published:** 2022-02-04

**Authors:** Sareena Sahab, Nigel Taylor

**Affiliations:** ^1^Agriculture Victoria Research, AgriBio, Centre for AgriBioscience, Bundoora, VIC, Australia; ^2^Donald Danforth Plant Science Center, St. Louis, MO, United States

**Keywords:** multiple left border technology, *Agrobacterium*-mediated transformation, vector backbone, transgenic cassava, transgene copy number

## Abstract

Imperfect T-DNA processing is common during *Agrobacterium*-mediated transformation, which integrates vector backbone sequences into the plant genome. However, regulatory restrictions prevent such transgenic plants from being developed for commercial deployment. The binary vector pCAMBIA2300 was modified by incorporating multiple left border (Mlb^®^) repeats and was tested in BY2 cells, tobacco, and cassava plants to address this issue. PCR analyses confirmed a twofold increase in the vector backbone free events in the presence of triple left borders in all three systems tested. Vector backbone read-through past the LB was reduced significantly; however, the inclusion of Mlbs^®^ did not effectively address the beyond right border read-through. Also, Mlbs^®^ increased the frequency of single-copy and vector backbone free events (clean events) twice compared to a single LB construct. Here, we briefly narrate the strength and limitations of using Mlb^®^ technology and reporter genes in reducing the vector backbone transfer in transgenic events.

## Introduction

Cassava (*Manihot esculenta* Crantz) is the largest sources of dietary calories in tropical and subtropical regions after rice and maize (Food and Agriculture Organization of the United Nations [FAO], 2008). Global cassava cultivation has increased approximately twofold since 1990, to reach 27 million hectares (Food and Agriculture Organization of the United Nations [FAO], 2019). However, cassava production is under pressure due to drought, weeds, pests, viral and bacterial diseases, and rapid post-harvest physiological deterioration (Patil and Fauquet, 2009; Naziri et al., 2014; Ekeleme et al., 2019; Orek et al., 2020). Genetic barriers such as high heterozygosity, irregular flowering, poor seed set and inbreeding depression acts as major bottlenecks for conventional breeding approaches in cassava (Elegba et al., 2021). Genetic transformation and genome editing can complement traditional or molecular breeding approaches to circumvent the challenges associated with the development of virus resistance and other valuable agronomic traits in cassava (Taylor et al., 2012a,b).

*Agrobacterium*-mediated transformation is the favored method for cassava genetic transformation using either somatic embryos or embryogenic callus as target tissues for transgene integration (Jørgensen et al., 2005; Taylor et al., 2012a). Despite progress, cassava transformation retains challenges such as genotype-dependent transformation methods, low regeneration rates, and changes in gene expression following embryogenesis and in some cases loss of resistance to Cassava Mosaic Disease (CMD) during tissue culture (Zainuddin et al., 2012; Ma et al., 2015; Beyene et al., 2017; Chauhan et al., 2018).

**FIGURE 1:**
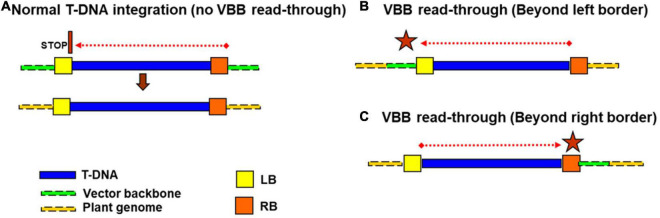
Different mode of vector backbone integrations **(A,B)** initiation of T-DNA transfer from RB **(C)** LB initiated T-DNA transfer.

As for other crop systems, *Agrobacterium*-mediated genetic transformation of cassava requires a transformation plasmid vector that houses the T-DNA consisting of the gene(s) of interest and a plant selectable marker bordered by two 25 bp imperfect repeats termed the left border (LB) and right border (RB) (Wang et al., 1984; Scheiffele et al., 1995). Outside the T-DNA region, the circular plasmid comprises a vector backbone (VBB) region that carries one or more bacterial selectable marker genes, replicons for bacterial multiplication, and other regulatory sequences required to maintain the plasmid in *E. coli* and *Agrobacterium*. In an ideal scenario, T-DNA delimited by the LB and RB is expected to be transferred from *Agrobacterium* to plant cell and then integrated into the plant genome intact, without any VBB sequences (Zupan and Zambryski, 1995; Gelvin, 2000; Tzfira and Citovsky, 2002). However, it has been known for some time that DNA sequences from the VBB can also be incorporated into the plant genome due to the imprecise nicking function of VirD2 nuclease, which causes the premature T-strand termination or read-through into vector backbone (Ramanathan and Veluthambi, 1995; van der Graaff et al., 1996; Kononov et al., 1997; Wenck et al., 1997). Initially, the RB was considered to be the unique initiation site for T-DNA transfer. However, Ramanathan and Veluthambi’s (1995) studies revealed that LB could also act as the T-DNA initiation site. Transfer of VBB sequences into the plant genome can result from one of two mechanisms; (1) DNA transfer is initiated at the RB proceeds across the T-DNA but fails to terminate at the LB, or (2) transfer is initiated at the LB toward the RB (Figure 1). Often vector backbone (VBB) read-through leads to either partial or complete integration of VBB (Ramanathan and Veluthambi, 1995; van der Graaff et al., 1996; Kononov et al., 1997; Yin and Wang, 2000). Numerous studies have confirmed the frequent occurrence of *Agrobacterium*-mediated VBB integration in a range of plant species such as rice, wheat, cotton, maize, and soybean (Kononov et al., 1997; De Buck et al., 2000; Afolabi et al., 2004; Lange et al., 2006; Podevin et al., 2006; Thole et al., 2007; Wu et al., 2007; Ye et al., 2008; Zhang et al., 2008; Wang et al., 2016). The frequency of VBB integration in transgenic events can be significant, with as many as 90% of regenerated transgenic plants reported to carry VBB sequences (Kuraya et al., 2004).

Integration of VBB sequences may exert undesirable negative effects in the transformants post-integration, such as *cis-*acting negative effect on gene expression from promoters (Artelt et al., 1991), illegitimate plasmid recombination, plasmid multimerization leading to the integration of large complexes comprising both exogenous and genomic DNA (Kohli et al., 1998; Salomon and Puchta, 1998; Müller et al., 1999). Furthermore, the process of multimerization often involves the capture of genomic DNA segments, resulting in multiple transgene copies. This is undesirable since high copy numbers may inhibit transgene expression and contribute to transgene silencing (Matzke et al., 1996). Additionally, very large transgenic loci can be meiotically unstable, leading to excision of the locus and loss of transgene expression in subsequent generations (Srivastava et al., 1996; Stoger et al., 1998). Presence of VBB sequences also raises concerns and complications for regulatory approval of Genetically Modified (GM) products (Fu et al., 2000).

Several strategies have been tested to produce VBB-free transgenic plants. These include: Binary vectors with small T-DNAs (Düring, 1994; Barrell and Conner, 2006), use of lethal genes in vector backbone (Hanson et al., 1999), tandem border repeats (Kuraya et al., 2004; Podevin et al., 2006), use of dual binary vector system, pCLEAN (Thole et al., 2007; Wang et al., 2016) and use of *Agrobacterium* chromosomes to launch T-DNA (Oltmanns et al., 2010).

Generation of elite cassava lines without VBB sequences would be highly desirable to develop GM enhanced varieties for commercialization to meet diverse challenges in cassava production. *Agrobacterium*-mediated transformation of cassava is routine in our laboratory (Taylor et al., 2012a,b; Beyene et al., 2017; Chauhan et al., 2018), with the goal to develop varieties for deployment to farmers in East Africa and Nigeria (Wagaba et al., 2017; Narayanan et al., 2019). Characterization of transgenic cassava plants revealed the presence of VBB sequences in 60–80% of the regenerants. This frequency negatively impacts efforts to develop high-quality transgenic plants readily acceptable to regulatory authorities for subsequent deployment to farmers. As a result, efforts were undertaken to reduce VBB integration and increase the frequency of quality transgenic plant lines. The current study reports on the outcome of deploying Pure Mlb^®^ (Multi-left border) technology in cassava transformation to reduce VBB integration, plus the use of visual scorable markers GFP and a gene encoding for phytoene synthase (PSY) as tools for early detection and elimination of plants carrying VBB sequences.

## Materials and Methods

### Transformation Vectors

#### Multiple Left Border Plasmids

**FIGURE 2:**
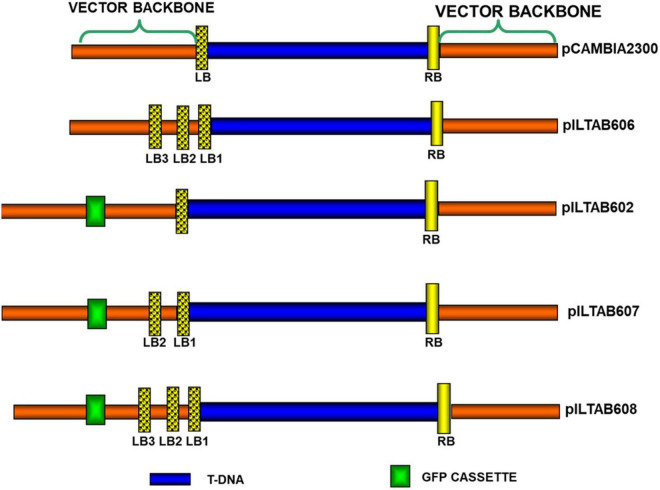
Schematic representation of vectors containing multiple left borders and reporter genes in the vector backbone.

Multiple left border (Mlb^®^) vectors were constructed following Kuraya et al. (2004) with a few modifications. The binary vector pCAMBIA2300^1^ was used in this study, and a unique restriction site *Nru*1 created through site-directed mutagenesis using the Stratagene™ kit (La Jolla, California). A sequence 5′-TCCCCGA-3′ located 17 bp distal from the existing LB repeat in pCAMBIA2300 was modified into 5′-TCGCGA-3′. The resulting plasmid was designated as p604 (Intermediate vector). Two sets of synthetic nucleotides (Supplementary Table 1) were annealed to generate sequences with two and three LB repeats, respectively. These short sequences were inserted independently into the *Nru*1 site of p604. The plasmids obtained were designated as p605 and p606, carrying two and three copies of LB repeats, respectively (Figure 2). Both plasmids were confirmed for the presence of additional LB repeats through sequencing.

#### Plasmids With a Visual Scorable Marker in the Vector Backbone

To test the utility of visual markers to screen for the presence of VBB, GFP (source: *Aequorea victoria*) and PSY (source: *Erwinia* sp. *crt*B gene) driven by an e35S promoter, were cloned independently in the VBB region of pCAMBIA2300 at 295 bp proximal to the left border (LB). The plasmids were designated as p602 and p603, respectively (Supplementary Figure 2). GFP cassette driven by e35S promoter were cloned at 294 bp proximal to LB of the multiple LB plasmids 605 and 606. The plasmids obtained were designated as p607 and p608 (Supplementary Data File 1).

### Plant Materials and Transformation of BY2 Cells and Cassava

Production of transgenic BY2 cells and tobacco (*Nicotiana tobaccum*) plants followed procedure reported earlier by Tse et al. (2004) and Clemente (2006), respectively, using different constructs as given in Figure 2. Cassava friable embryogenic callus (FEC) were derived from organized embryogenic structures (OES) induced from immature leaf lobes of *in vitro* cassava shoot cuttings of variety 60444 as descried by Taylor et al. (2012a). FEC target tissues were transformed with *A. tumefaciens* strain LBA4404 using the gene constructs shown in Figure 2, and plants regenerated per Taylor et al. (2012a) (Supplementary Figure 1).

### Visualization of Transgenic GFP and Phytoene Synthase Expression in Cells

GFP expressing FEC and regenerating somatic embryos are visualized using a Nikon SMZ 1500 stereomicroscope, UV illumination, and an HQ-FTIC-Long Pass filter set. Brightfield settings were used to visualize phytoene synthase (PSY) gene expression.

### PCR Analysis for Detection of Vector Backbone Sequences

Genomic DNA was extracted from BY2 cells, tobacco plants, and cassava leaves following Dellaporta et al. (1983) with the following modifications. Approximately 100 mg of sample was ground in 350 μl extraction buffer [Tris-HCl (0.01 M), EDTA (0.05 M), and NaCl (0.5 M)]. The final volume was made up to 450 μl using100 μl of sterile distilled water and to this 1.0 μl of β-Mercaptoethanol added at the time of DNA extraction using MP Fast Prep at 4.0 M/S for 20 s. One microliter of RNAse was added and mixed by inverting. The plant extract was homogenized in 100 μl of 10% SDS and incubated at 65°C for 10 min, followed by 250 μl of potassium acetate (5 M) (Sambrook and Russell, 2001). Samples were incubated on ice for at least 5 min and centrifuged at 14,000 revs for 5 min. The supernatant was clarified by passing through a Miracloth into a 1.5 ml tube containing 500 μl isopropanol and incubated at –20°C for 30 min. The samples were then centrifuged at 14 000 for 10 min, the supernatant discarded, the pellet washed with 1 ml of 70% ethanol, and the supernatant removed by centrifuging 14,000 for 5 min. Pellets were dried for 30 min and the DNA dissolved in 100 μl of sterile water. Exactly 2 μl of DNA was used for PCR reactions.

**FIGURE 3:**
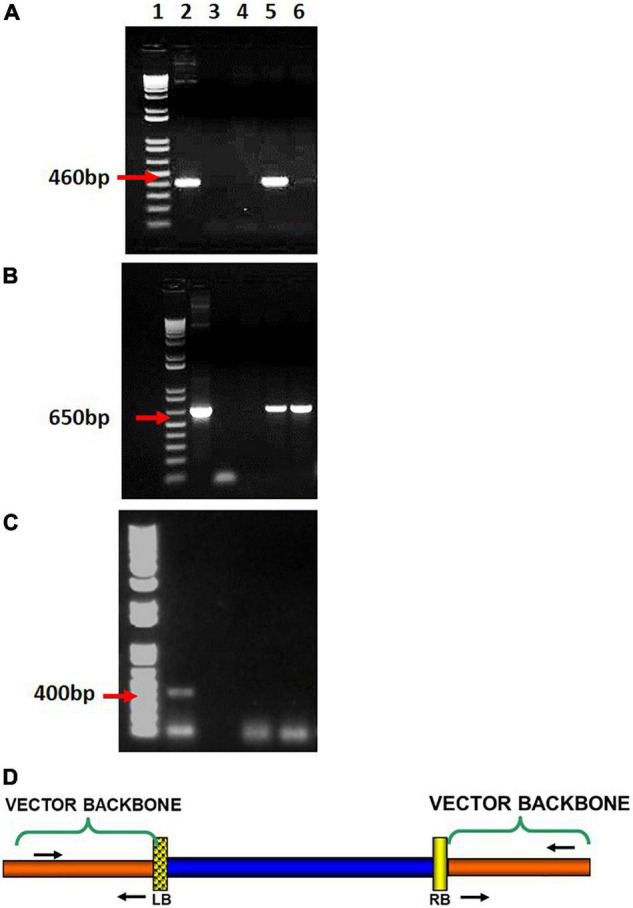
PCR based detection of vector backbone read-through, **(A)** beyond left border, **(B)** beyond right border, **(C)** *Agrobacterium* carryover tested in transgenic cassava plants (sample gel images). (Lanes: 1-1 kb + ladder; 2-Positive plasmid control; 3- Negative control; 4–6 DNA sample (Transgenic tissue) **(D)** primer locations to detect beyond LB and RB read-throughs.

Putative transgenic events were confirmed initially by performing a PCR for detection of the *npt*II gene and the *pic*A gene (Kononov et al., 1997) to detect any possible *Agrobacterium* contamination. Events positive for *npt*II and negative for agro contamination were further screened for presence of VBB. Sequences of primer pairs and their annealing temperature are shown in Supplementary Tables 1, 2 and their respective positions within the plasmid detailed in Figure 3D. All PCR reactions were performed using a Bio-Rad^®^ thermocycler, model “ICycler.” PCR conditions consisted of an initial denaturation period of the 30 s at 98°C followed by another 10 s at 98°C (for annealing temperature settings refer to Supplementary Table 2), an extension temperature of 72°C for 45 s and a final extension period at 72°C for 10 m. The completed reaction was held at 4°C. Plasmid DNA was used as positive controls, and DNA isolated from non-transgenic cv 60444 was used as the negative control.

### Transgene Copy Number Analysis

**FIGURE 4:**
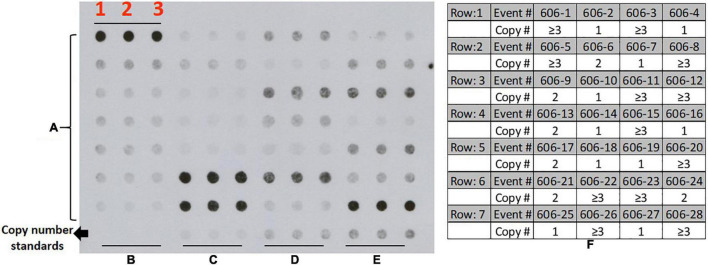
Transgene copy number assessment in cassava using Dot Blot technique (representative image); **(A)** DNA samples from transgenic cassava plants generated from the current study (28 samples from construct p606 (pCAMBIA2300 + 3LB; rows 1–7) row: 8, **(B)** zero copy; **(C)** single copy; **(D)** double copy; **(E)** triple copy; each sample loaded in triplicate for comparison; **(F)** copy number estimates for 28 events represented in the dot blot image.

Genomic DNA was isolated from young *in vitro* leaves using DNeasy plant mini kit (Qiagen, Hilden, Germany), and copy numbers estimated by dot blot analysis (Bhatnagar et al., 2010; Taylor et al., 2012a). One hundred nanograms of DNA was blotted in triplicate unto Hybond-N + nylon membrane (GE Healthcare, Piscataway, NJ). One, two, and triple copy number events previously tested transgenic cassava events were used as reference standards for low and high T-DNA copy number (Taylor et al., 2012a). Membranes for dot blots were hybridized to a Digoxigenin (DIG)-labeled (Roche, Indianapolis, United States) probe generated by PCR amplification of a 323 bp fragment of the CaMV 35S promoter (primer pair: F-cacatcaatccacttgctttgaag and R-catggtggagcacgacact.) and used to test all putative transformants derived from all the vectors tested in this study. Hybridization of membrane-bound DNA to DIG-labeled probes followed by washing and detection using CDP-Star (Roche, Indianapolis, United States) was performed per manufacturer instructions. DNA-probe hybridization was visualized by exposing the membrane to X-ray film (Figure 4). The developed films were scanned and saved as Tiff files. Scanned and saved X-ray films were analyzed using open-source Image J^2^ software version 1.36b (Schneider et al., 2012)^3^ as described by Taylor et al. (2012a). Image J (see footnote 2) elliptical selection tool was used to measure the intensity of the signal, and data were automatically exported as an excel file. Copy number for each transgenic line was extrapolated from the average of the triplicated dots based on a slope calculated using the reference standards.

### Statistical Analysis

Each experiment was performed twice, and data generated analyzed using Microsoft Excel Version. 2102 (Supplementary Data File 2).

## Results

### Detection of Vector Backbone Integration Using Visual Scorable Marker Gene

**FIGURE 5:**
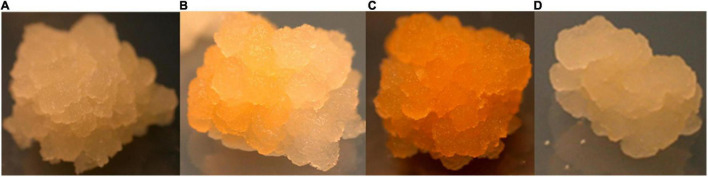
Phytoene synthase expression in BY2 cells **(A)** colorless callus **(B)** partially colored callus **(C)** fully colored callus **(D)** colorless (callus derived from control plasmid-pCambia2300).

**FIGURE 6:**
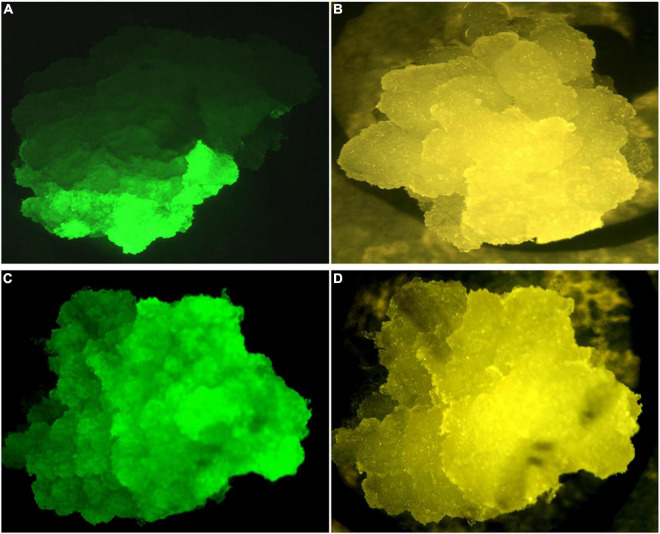
GFP expression in BY2 cells **(A)** partial GFP expression, **(C)** whole callus expressing GFP under GFP filter settings **(B,D)** Brightfield filter.

The performance of the constructs was initially assessed in BY2 cells due to the ability to generate transgenic events within a short period (approximately 8–10 weeks). Independent putative transgenic BY2 cell lines transformed with p602 (GFP in VBB) and p603 (Phytoene Synthase; PSY in VBB) were screened for reporter gene expression 6 weeks post *Agrobacterium* co-culture. Reporter gene expressions were similar across both reporter gene systems with approximately 35% of the callus lines expressed GFP or PSY from the VBB located expression cassettes (data not shown). As shown in Figure 5, scoring based on PSY gene expression resulted in a range of expression patterns, and this was considered a challenge in its deployment to screen for the presence of VBB presence. Conversely, GFP-based scoring enabled thorough screening for the VBB read-throughs (Figure 6) with less ambiguity than PSY. Therefore, GFP within the VBB was used for further studies in cassava.

VBB read-through in cassava FEC was detected using a reporter gene (GFP) based approach and compared with PCR-based assays. Reporter gene-based scoring as summarized in Table 1 showed a preliminary indication on the effect of additional LB repeats in reducing VBB read-through, and it enabled in detecting VBB insertion frequencies as 37, 23, and 15% for vectors p602 (1LB + GFP in VBB), p607 (2LB + GFP in VBB), and p608 (3LB + GFP in VBB) respectively. However, the frequencies were 50% or less than the frequency of VBB integration obtained through PCR analysis (Table 1). The results obtained thus indicated that the use of marker genes could not help addressing this issue on its own, and it still required detailed molecular analyses to confirm the overall VBB integration frequency. However, in some of the callus units screened, the expression of the GFP was found to be partial. Under such circumstances, it is still worthwhile using the marker genes to detect the VBB sequences as it is possible to separate the portion of callus lacking any expression which may be free of VBB sequences.

**TABLE 1:** Comparison between VBB detection through PCR and GFP expression in transgenic cassava callus.

Constructs tested	*No. of explants screened for GFP expression	No. of explants positive for GFP gene based on PCR analyses (%)	No. of explants positive for GFP expression and PCR analyses (%)	No. of explants PCR positive for GFP gene but lacking GFP expression (%)
pILTAB602 (1LB + GFP in VBB)	100	80 (80)	37 (37)	43 (43)
pILTAB607 (2LB + GFP in VBB)	80	49 (61)	19 (23)	30 (38)
pILTAB608 (3LB + GFP in VBB)	90	42 (47)	14 (15)	28 (31)

Explants- FEC, friable embryogenic callus; * Data obtained from 2 independent experiments.

Thus, the present study revealed the strength and weakness of using a visual scorable marker in detecting the VBB sequences, and therefore we concluded that molecular analyses should be used as the ultimate screening method. Similar trends were observed in BY2 callus lines and transgenic tobacco lines (Supplementary Table 3).

### Effect of Pure Multiple Left Border on Vector Backbone Integration in Transgenic Cassava Plants

**FIGURE 7:**
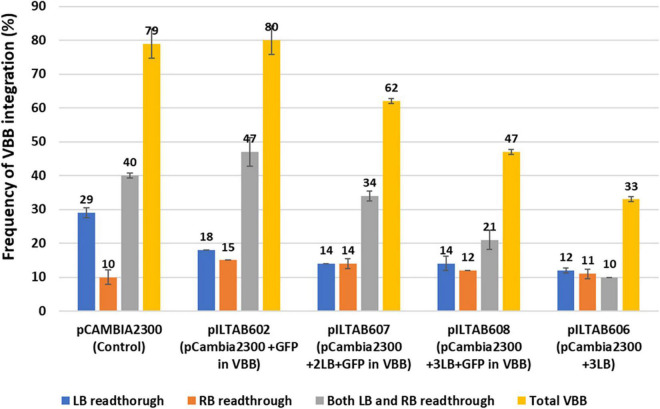
Frequency of vector backbone integration in transgenic cassava plants; Data obtained from two independent experiments.

The independent lines positive for the NPTII gene from test constructs were tested for vector backbone sequences. The VBB read-through in transgene loci was detected using primer sets located outside the left and right T-DNA borders (Figure 3 and Supplementary Table 2). The results of vector backbone integration in the transgenic cassava lines generated from each vector are shown in Figure 7. For pCAMBIA2300 (control vector with 1LB), 79% of the tested lines contained VBB sequences, of which 40% of the lines had VBB integration flanking both LB and RB. A similar trend was observed for vector p602 (pCAMBIA2300 + GFP in the VBB). For p606 (pCAMBIA2300 + 3LB), only 34% of the tested lines showed presence for VBB integration, of which only 11% of the lines showed for the presence for both beyond left and right border read-throughs. The presence of triple LB showed an approximately twofold reduction in the insertion of VBB DNA compared to single LB constructs. Furthermore, the frequencies of transgenic lines containing VBB sequences were 80, 61, and 47% for p602 (pCAMBIA2300 + GFP in VBB), p607 (pCAMBIA2300 + 2LB + GFP in VBB), and p608 (pCAMBIA2300 + 3LB + GFP in VBB), respectively (Figure 7). Furthermore, the frequencies of transgenic lines containing VBB sequences flanking RB were 10, 15, 14, 12, and 11% for pCAMBIA2300 (control vector with 1LB), p602, p607, p608, and p606, respectively. The results obtained thus indicated that the vector backbone read-throughs were more frequently linked to the LB than to the RB, and LB stacking did not address the issues beyond RB vector backbone integrations. Similar trends were observed in BY2 callus lines and transgenic tobacco lines (Supplementary Tables 4, 5).

### Presence of Vector Backbone Read Through and Its Impact on Transgene Copy Number in Transgenic Cassava Events

This study investigated the integration of VBB sequences and their impact on T-DNA copy numbers in the plant genome. Dot blot hybridization was used to estimate the T-DNA copy number of plants derived from the five constructs pCAMBIA2300, p602, p607, p608, and p606. Amongst the events tested from pCAMBIA2300, p602, p607, p608, and p606, 10% (5/48), 9% (5/55), 17% (10/58), 30% (20/67), and 34% (26/39) were single copy and VBB free events, respectively (Table 2). The incorporation of triple LB repeats resulted in a threefold increase in clean events compared to single and double LB constructs. Furthermore, data summarized in Table 3 shows the frequency of VBB integration and events with high and low copy numbers from constructs p602, p607, and p608. Correlation analysis was carried out to confirm the relationship between VBB integration and multiple T-DNA integration. A correlation coefficient of the data revealed that the number of VBB-integrated events showed a significant negative correlation (*r* = –0.052414) for low T-DNA copy number events and a significant positive correlation (*r* = 0.973985) for high T-DNA copy number events (Table 4). These results show a significant correlation for VBB integration amongst events with multiple T-DNA integration. The positive correlation for high copy number and events with VBB indicates that the higher the copy number of T-DNA integrated into the plant genome, the lower the number of VBB-free events recovered and vice versa.

**TABLE 2:** Frequency of VBB free, single T-DNA copy in transgenic cassava plants.

Constructs tested	No. of NPTII positive events tested	No. of VBB free events (%)	No. of single copy events (%)	No. of clean events* (%)
pCAMBIA2300 (Control)	48	10 (21)	12 (25)	5 (10)
p602 (1LB + GFP in VBB)	55	11 (20)	11 (20)	5 (9)
P607 (2LB + GFP in VBB)	58	23 (39)	22 (38)	10 (17)
p608 (3LB + GFP in VBB)	67	36 (53)	29 (43)	20 (30)
p606 (3LB)	78	51 (66)	39 (50)	26 (34)

*Clean events: Single copy and VBB free events.

**TABLE 3:** Frequency of vector backbone integration and T-DNA copy number (High and low copy events) in transgenic cassava plants.

	No. of plants tested	No. of plants with VBB integration (%)	No. of low copy plants with VBB (%)	No. of high copy plants with VBB (%)
pILTAB602 (1LB + GFP in VBB)	55	44 (80)	10 (19)	34 (61)
pILTAB607 (2LB + GFP in VBB)	58	36 (62)	15 (26)	21 (36)
pILTAB608 (3LB + GFP in VBB)	47	31 (47)	13 (19)	19 (28)

*VBB, vector backbone, low defined as 1-2 T-DNA copies, high defined as 3 or more T-DNA copies.*

**TABLE 4:** Correlation coefficients for events with VBB presence and transgene copy number.

	VBB integration (%)	Low copy with VBB (%)	High copy with VBB (%)
VBB integration (%)	1		
Low copy with VBB (%)	–0.052414242	1	
High copy with VBB (%)	0.972128239	–0.28508085	1

*Data from two independent experiments; p = 0.01.*

## Discussion

The exclusion of non-essential sequences from transformation vectors and generation of low T-DNA copy GM plants are desirable to achieve timely regulatory approval and public acceptance of crop biotechnology. Event characterization requires assessment of transgene copy number, thorough screening across the junctions of gene cassettes inserted into the plant genome and detection of the molecular consequences of transgene insertion, including small and large-scale deletions, rearrangements of plant DNA, gene disruption and superfluous DNA integration (Jorgensen et al., 1996; Stam et al., 1997; Latham et al., 2006; Gelvin, 2012). Assays include PCR- and sequencing-based approaches to ensure that candidate events meet regulatory and performance requirements. It is worthwhile, therefore, to employ strategies upfront to increase the frequency of quality events and reduce the downstream costs associated with screening a large number of primary transgenic events.

Analysis of transgenic cassava plants generated in our laboratory indicated a VBB integration frequency reached almost 80% (Figure 7). A primary goal of our laboratory is to develop genetically modified cassava plants that meet requirements for regulatory approval and release to farmers (Taylor et al., 2012a). The present study was undertaken, therefore, to improve frequencies for the recovery of quality transgenic events, most especially to achieved reduction in the occurrence of VBB integration. We report the outcomes of two strategies (i) use of reporter genes in VBB and (ii) use of multiple LB (Pure Mlb^®^) to address the high frequency of VBB insertions in transgenic cassava events.

Preliminary studies using GFP and PSY expression cassettes placed within the VBB past the LB enabled early detection of VBB presence in a non-destructive manner. However, PCR-based screening was required to detect the total VBB integration events in both BY2 cells and cassava using the reporter genes in this manner. Similar results for use of a marker gene within the VBB were reported by Kononov et al. (1997), where use of *gus*A in the VBB detected 19% of the VBB sequences through GUS staining. However, a lack of power for this approach was confirmed by PCR analyses which revealed that 74% of the GUS negative plants carried a portion of the *gus*A and integrated VBB sequence.

We therefore investigated the use of multiple left order sequences. After *Agrobacterium*-mediated transformation and recovery of transgenic cassava plants, integration of backbone sequences detected by PCR were 79, 80, 62, 47, and 33% for pCAMBIA2300 (control vector with 1LB), p602 (pCAMBIA2300 + GFP in VBB), p607 (pCAMBIA2300 + 2LB + GFP in VBB), p608 (pCAMBIA2300 + 3LB + GFP in VBB), and p606 (pCAMBIA2300 + 3LB), respectively (Figure 7). Data demonstrated that incorporating triple LB repeats could significantly reduce VBB integration frequency into the plant genome compared to constructs carrying single LB in cassava (Table 3 and Figure 7). However, variations in frequency were observed between the three different systems tested; BY2 cells had a slightly higher VBB integration frequency than tobacco and cassava transgenic events. The higher VBB frequency observed could have resulted from the chimeric or mosaic nature of BY2 callus compared to whole plants in tobacco and cassava. The frequency of VBB integration observed was slightly higher than reported by Kuraya et al. (2004) using multiple LB strategies. The outcomes could have resulted from the difference in the tissue system, the type of transformation vector used (binary plasmid instead of co-integrate vector system), and the inclusion of RB read-through assays in the events tested in the present study. Similar reductions in VBB transfer were reported using multiple LB strategies (Huang et al., 2004; Kuraya et al., 2004; Podevin et al., 2006; Wang et al., 2016).

In the present study, we observed different modes of VBB read-through across all the three systems studied (Figure 7). T-strand initiation and termination were initially considered unidirectional from right to the left border (Wang et al., 1984; Horsch and Klee, 1986; Jen and Chilton, 1986; Joersbo and Okkels, 1996). However, the transfer of backbone sequences could be due to the failure to terminate the T-strand at the left border (read through) or due to the initiation of a T-strand at the left border. Furthermore, initiation of T-DNA transfer at LB and RB has been reported leading to the VBB read-throughs beyond LB and RB (Miranda et al., 1992; Wenck et al., 1997; De Buck et al., 2000; Yin and Wang, 2000; Podevin et al., 2006).

The inclusion of Mlbs^®^ resulted in a twofold reduction in VBB integration across the LB compared to the control plasmid (Figure 7, Table 2, and Supplementary Tables 4, 5). However, it had no effect on read-through across RB. A previous report by Kuraya et al. (2004) did not assess for VBB read-through across RB, which could account, therefore, for the higher frequency of VBB free transgenic events described in their study.

### Correlation Between Transgene Copy Number and Vector Backbone Integration

Copy number analyses of transgenic cassava plants revealed that an increase in the VBB free plants increased the proportion of single-copy plants from 25 to 50% in triple LB constructs. Importantly, this resulted in a threefold increase in single-copy, VBB free events (quality events) compared to the control construct (Table 2). Similar observations have been made by Kuraya et al. (2004), Vain et al. (2004), and Ye et al. (2008). A probable reason could be that termination of VBB read-through reduces the whole VBB integration, which, in turn, reduces incorporation of additional T-DNA copies in the transgenic events as concatamers. This is evident from the present study, where we could group the low copy (one and two copy events) and multi-copy events based on VBB read-through (Table 3), with low copy events found to have higher frequencies of VBB free integrations. High copy events (3 or more transgene copies) events were found to have higher frequencies of events incorporating both LB and RB read-throughs. Complex or multigene transgene integrations often result from the LB’s failure to terminate T-DNA integration, thus allowing repeated integration of the T-DNA.

In conclusion, the data presented here reveals that incorporating Mlbs^®^ reduces the VBB integration in transgenic BY2 cells, tobacco and cassava. Also, the correlation studies between copy number and VBB indicate that increasing the frequency of VBB free transgenic events results in a proportionate increase in the low-copy events. Based on this study, Mlb^®^ technology (Triple LB) was incorporated in the routine transgenic cassava pipeline (Beyene et al., 2017; Narayanan et al., 2019, 2021).

Recent advances in precision genome engineering approaches using site-specific nucleases such as ZFN’s and CRISPRs have offered improved and sustainable platforms to address some of the challenges in conventional transgenesis methods (Ainley et al., 2013; Voytas and Gao, 2014; Kumar et al., 2016). CRISPR/Cas9 has been used to generate mutants in Cassava successfully (Odipio et al., 2018; Gomez et al., 2019; Mehta et al., 2019; Veley et al., 2021). Thus, these precision engineering tools, along with the clean transgene technology such as use of Pure Mlb^®^ demonstrated in this study, will benefit the production of clean genetically modified cassava plants and thus simplify the biosafety evaluation process and facilitate the future commercialization to meet diverse challenges in a global context.

## Data Availability Statement

The original contributions presented in the study are included in the article/Supplementary Material, further inquiries can be directed to the corresponding author/s.

## Author Contributions

SS: performed all outline experiments and manuscript preparations. NT: experimental designs, cassava transformation, and manuscript review. Both authors contributed to the article and approved the submitted version.

## Conflict of Interest

The authors declare that the research was conducted in the absence of any commercial or financial relationships that could be construed as a potential conflict of interest.

## Publisher’s Note

All claims expressed in this article are solely those of the authors and do not necessarily represent those of their affiliated organizations, or those of the publisher, the editors and the reviewers. Any product that may be evaluated in this article, or claim that may be made by its manufacturer, is not guaranteed or endorsed by the publisher.

## Footnotes

1
http://www.cambia.org

2
https://imagej.nih.gov/ij/

3
http://rsbweb.nih.gov/ij/
