# Supplementary material for: Studies on Pure Mlb® (Multiple Left Border) Technology and Its Impact on Vector Backbone Integration in Transgenic Cassava
Source: Front Plant Sci. 2022 Feb 4;13:816323. doi: 10.3389/fpls.2022.816323 (PMC8855067; doi:10.3389/fpls.2022.816323)
Supplement: Supplementary file 1 [file Data_Sheet_1.PDF]

Supplementary data file.1

(a) Steps involved in creating vectors with additional LB sequences

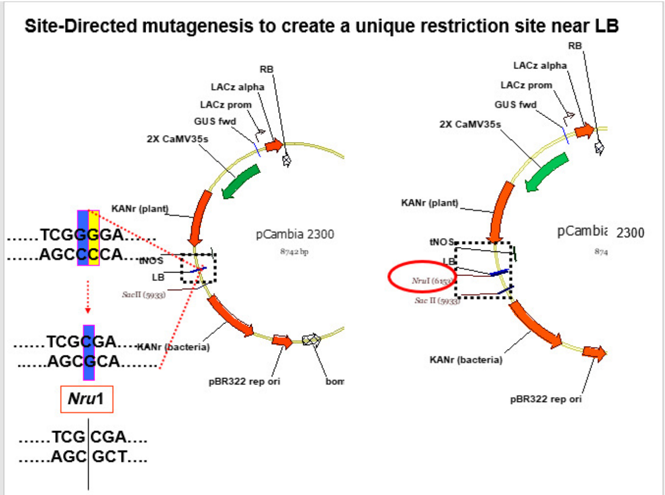

(b) and (c) pCAMBIA2300 vector cloned with two and three LB repeats, respectively.

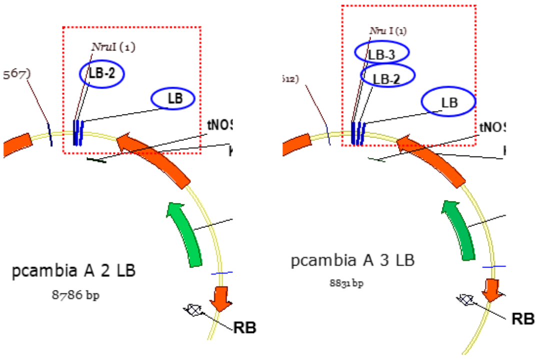

(b) (c)

(d) and (e) pCAMBIA2300 vector cloned with two and three LB repeats and GFP cassette in the VBB, respectively.

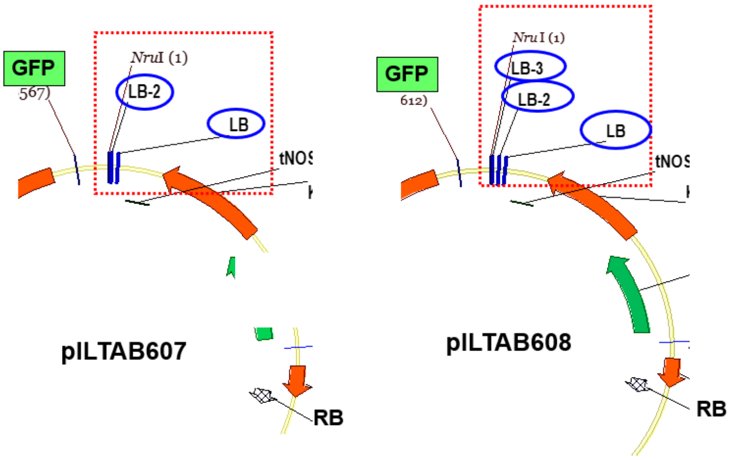

(d) (e)
