# Supplementary material for: Studies on Pure Mlb® (Multiple Left Border) Technology and Its Impact on Vector Backbone Integration in Transgenic Cassava
Source: Front Plant Sci. 2022 Feb 4;13:816323. doi: 10.3389/fpls.2022.816323 (PMC8855067; doi:10.3389/fpls.2022.816323)
Supplement: Supplementary file 2 [file Data_Sheet_2.PDF]

Statistical analysis:

Supplementary Data file. 2

Statistical analysis on the effect of Pure Mlb® technology in reducing vector backbone integration:

Data analysis performed using Microsoft Excel Data analysis package

Treatments: No. of LB repeats tested	Vector detail	Frequency of VBB free events (%)
1	pCAMBIA2300 (Control)	21
1	pILTAB602	20
2	PILTAB607	39
3	pILTAB608	53
3	pILTAB606	66

Data analyzed from two independent experiments (Total of 306 events tested)

SUMMARY OUTPUT

Regression Statistics	
Multiple R	0.972934706
R Square	0.946601942
Adjusted R Square	0.928802589
Standard Error	0.26682843
Observations	5

ANOVA

	df	*SS*	*MS*	*F*	*Significance F*
Regression	1	3.786407767	3.786407767	53.18182	0.005323312
Residual	3	0.213592233	0.071197411		
Total	4	4			

α=0.05

	Coefficients	*Standard Error*	*t Stat*	*P-value*
Intercept	0.067961165	0.290565649	0.233892635	0.830121
Frequency of VBB free events (%)	0.048543689	0.006656581	7.292586522	0.005323

Addition of multiple LB repeats had a significant effect in reducing the vector backbone insertion in transgenic cassava events tested (F value <α=0.05)
