# Supplementary figures and images for: Studies on Pure Mlb® (Multiple Left Border) Technology and Its Impact on Vector Backbone Integration in Transgenic Cassava
Source: Front Plant Sci. 2022 Feb 4;13:816323. doi: 10.3389/fpls.2022.816323 (PMC8855067; doi:10.3389/fpls.2022.816323)

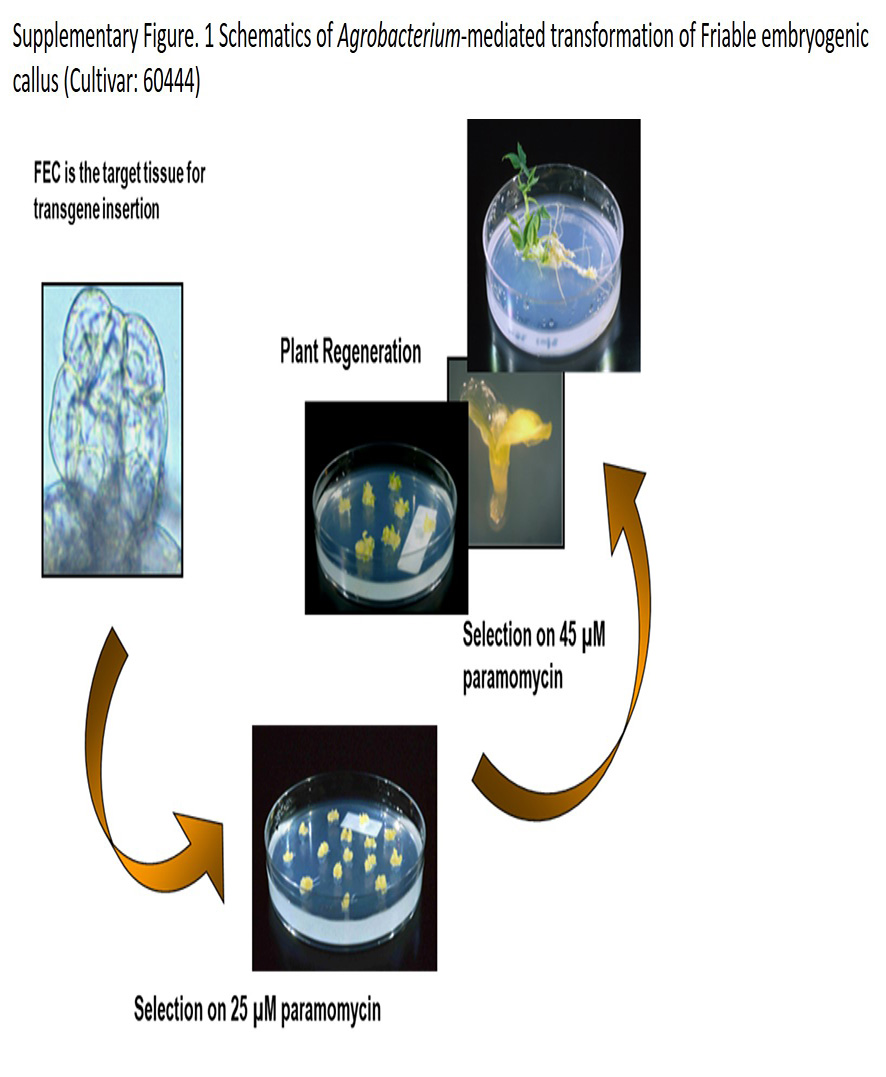

Supplement: Supplementary file 3 [file Image_1.JPEG]

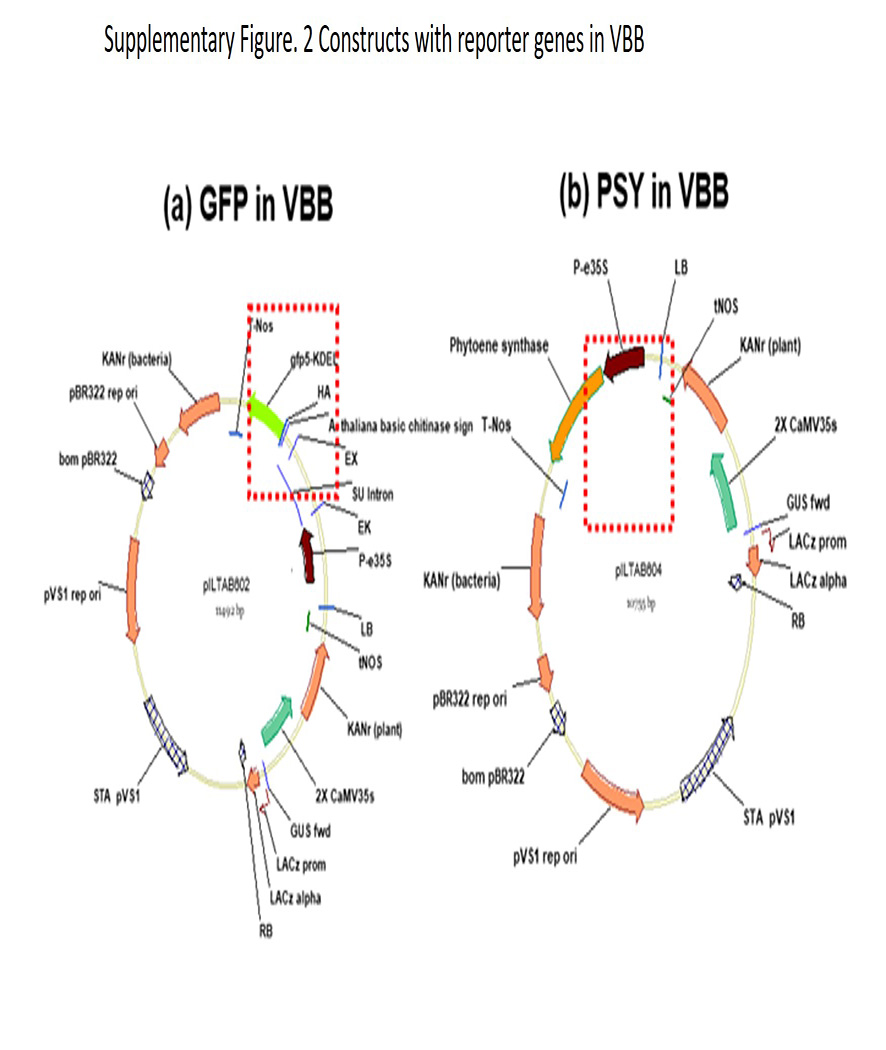

Supplement: Supplementary file 4 [file Image_2.JPEG]

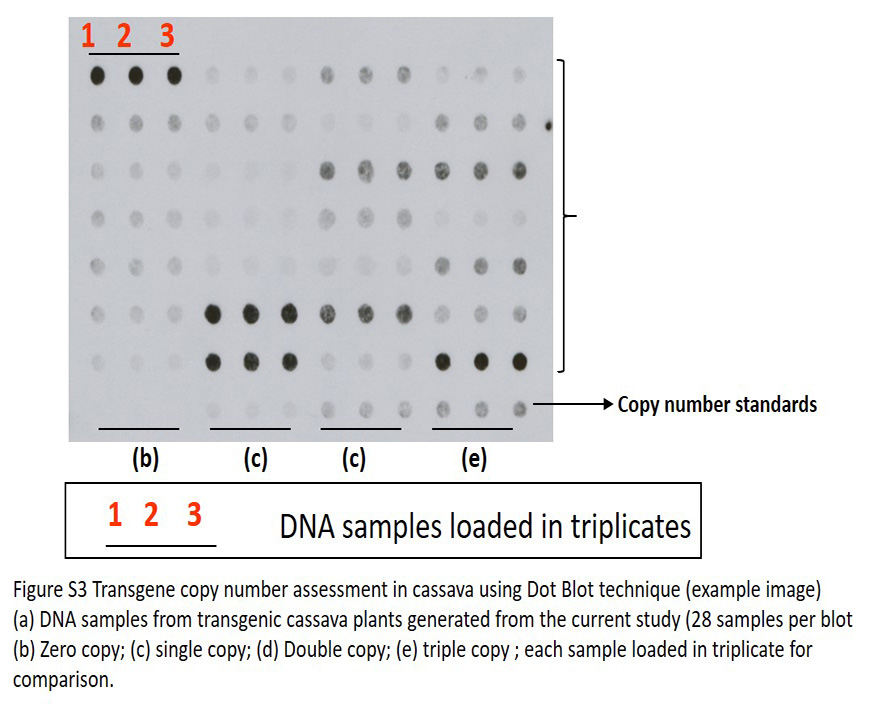

Supplement: Supplementary file 5 [file Image_3.JPEG]
