# Supplementary material for: Studies on Pure Mlb® (Multiple Left Border) Technology and Its Impact on Vector Backbone Integration in Transgenic Cassava
Source: Front Plant Sci. 2022 Feb 4;13:816323. doi: 10.3389/fpls.2022.816323 (PMC8855067; doi:10.3389/fpls.2022.816323)
Supplement: Supplementary file 6 [file Table_1.DOCX]

Table S1 Sequence of synthetic nucleotides used to generate addition LB sequences

|  | **Synthetic sequences** |
| --- | --- |
| Set 1  (1LB) | 5’-gttacaccacaatatatcctgccaccagccaacagctcg-3’;  5’-cgagctgttggctggctggtggcaggatatattgtggtgtaaac-3’ |
| Set 2  (2LB) | 5’gagctgttggctggctggtggcaggatatattgtggtgtaaacacgagctgttggctggctggtggcaggatatattgtggtgtaaaca-3’;  5’-gctcgacaacccgaccgaccaccgtcctatataacaccacatttgtgctcgacaaccgaccgaccaccgtcctatataacaccacatttgt-3’ |
