# Supplementary material for: Studies on Pure Mlb® (Multiple Left Border) Technology and Its Impact on Vector Backbone Integration in Transgenic Cassava
Source: Front Plant Sci. 2022 Feb 4;13:816323. doi: 10.3389/fpls.2022.816323 (PMC8855067; doi:10.3389/fpls.2022.816323)
Supplement: Supplementary file 7 [file Table_2.DOCX]

Table S2 Primer pairs for detection of transgene and presence of *Agrobacterium* contamination and VBB sequences

| Primer pair | Sequence | *T_A_°C |
| --- | --- | --- |
| *npt*II F  *npt*II R | 5'-tcagaagaactcgtcaagaaggcg-3'  5'-atgattgaacaagatggattgcac-3' | 59 |
| *pic*A F  *pic*A R | 5'-atgccgcatgaggctcgtcttcgac-3'  5'-gacgcaacgcatcctcgatcagct-3' | 55 |
| LB F  LB R | 5'-ggttcaaacccggcagcttagttg-3'  5'-gggttcctatagggtttcgctcatg-3' | 58 |
| RB F  RB R | 5'-cagcctgaatggcgaatgctagag-3'  5'-cctagaatgcatgaccaaaatcc-3' | 57 |

*T_A_ Annealing temperature; (all PCR amplifications run at 30 cycles)
