# Supplementary material for: Studies on Pure Mlb® (Multiple Left Border) Technology and Its Impact on Vector Backbone Integration in Transgenic Cassava
Source: Front Plant Sci. 2022 Feb 4;13:816323. doi: 10.3389/fpls.2022.816323 (PMC8855067; doi:10.3389/fpls.2022.816323)
Supplement: Supplementary file 8 [file Table_3.DOCX]

Table S3 Comparison between VBB detection through PCR and GFP expression

|  | Constructs tested | PCR analyses  % | GFP expression  % |
| --- | --- | --- | --- |
| BY2  (callus lines) | pILTAB602  (1LB + GFP in VBB) | 91 | 34 |
|  | pILTAB607  (2LB + GFP in VBB) | 67 | 22 |
|  | pILTAB608  (3LB + GFP in VBB) | 57 | 15 |
| Tobacco | pILTAB602  (1LB + GFP in VBB) | 79 | 36 |
|  | pILTAB607  (2LB + GFP in VBB) | 47 | 25 |
|  | pILTAB608  (3LB + GFP in VBB) | 32 | 18 |

Data from two independent experiments; VBB-Vector backbone; LB-Left Border
