# Supplementary material for: Studies on Pure Mlb® (Multiple Left Border) Technology and Its Impact on Vector Backbone Integration in Transgenic Cassava
Source: Front Plant Sci. 2022 Feb 4;13:816323. doi: 10.3389/fpls.2022.816323 (PMC8855067; doi:10.3389/fpls.2022.816323)
Supplement: Supplementary file 9 [file Table_4.DOCX]

Table S4 Frequency of VBB in transgenic BY2 callus lines

| Constructs tested | No. of lines tested | VBB integration frequency  % | | | Total  VBB  % |
| --- | --- | --- | --- | --- | --- |
|  |  | LB+RB- | LB-RB+ | LB+RB+ |  |
| pILTAB602  (1LB + GFP in VBB) | 90 | 48 | 17 | 26 | 91 |
| pILTAB607  (2LB + GFP in VBB) | 95 | 31 | 14 | 22 | 67 |
| pILTAB608  (3LB + GFP in VBB) | 100 | 23 | 16 | 18 | 57 |

Data from two independent experiments; VBB read-through-past LB alone (LB+RB-), VBB read-through-past RB alone (LB-RB+), VBB read-through-past LB and RB (LB+RB+) VBB –Vector backbone; LB-Left Border; RB-Right border
