# Supplementary material for: Studies on Pure Mlb® (Multiple Left Border) Technology and Its Impact on Vector Backbone Integration in Transgenic Cassava
Source: Front Plant Sci. 2022 Feb 4;13:816323. doi: 10.3389/fpls.2022.816323 (PMC8855067; doi:10.3389/fpls.2022.816323)
Supplement: Supplementary file 10 [file Table_5.DOCX]

Table S5 Frequency of VBB in transgenic tobacco lines

| Constructs tested | No. of lines tested | VBB integration frequency  % | | | Total  VBB  % |
| --- | --- | --- | --- | --- | --- |
|  |  | LB^+^RB^-^ | LB^-^RB^+^ | LB^+^RB^+^ |  |
| pILTAB602  (1LB + GFP in VBB) | 48 | 44 | 17 | 19 | 80 |
| pILTAB607  (2LB + GFP in VBB) | 50 | 21 | 16 | 10 | 47 |
| pILTAB608  (3LB + GFP in VBB) | 47 | 13 | 17 | 2 | 32 |

Data from two independent experiments; VBB read-through-past LB alone (LB+RB-), VBB read-through-past RB alone (LB-RB+), VBB read-through-past LB and RB (LB+RB+) VBB –Vector backbone; LB-Left Border; RB-Right border
